# Theaflavin-3,3′-digallate triggers apoptosis in osteosarcoma cells via the caspase pathway

**DOI:** 10.7150/jca.111718

**Published:** 2025-05-27

**Authors:** Yat-Yin Law, Yi-Hsien Hsieh, Yih-Shou Hsieh, Yi-Hsun Lee, Chia-Ling Tang, Chia-Yi Lee, Shun-Fa Yang, Shu-Chen Chu, Pei-Ni Chen

**Affiliations:** 1 Institute of Medicine, Chung Shan Medical University, Taichung, Taiwan; 2 School of Medicine, Chung Shan Medical University, Taichung, Taiwan; 3 Department of Orthopedics, Chung Shan Medical University Hospital, Taichung, Taiwan; 4 Department of Medical Research, Chung Shan Medical University Hospital, Taichung, Taiwan; 5 Institute and Department of Food Science, Central Taiwan University of Sciences and Technology, Taichung, Taiwan

**Keywords:** osteosarcoma, theaflavin-3,3'-digallate, reactive oxygen species, caspase, apoptosis

## Abstract

Osteosarcoma is a cancer associated with a guarded prognosis. Various compounds can induce apoptosis in osteosarcoma cells. Theaflavin-3,3′-digallate (TF3) has been demonstrated to alter cell growth and induce apoptosis in various cancer cells. The present study investigated the apoptotic effect of TF3 on osteosarcoma cells. It further explored key apoptotic pathways activated by TF3. Viability of 143B and U2OS osteosarcoma cells after TF3 treatment was assessed. The effects of TF3 on key apoptotic pathways were analyzed. Furthermore, a xenograft mouse model of osteosarcoma was established for *in vivo* experiments. The results indicated that TF3 significantly reduced the viability of 143B and U2OS cells. Western blotting revealed that TF3 upregulated the expression of cleaved caspase-3 and cleaved caspase-9 in osteosarcoma cells. In addition, TF3 increased the levels of phosphorylated histone H2Ax, Bax, Bak1, and cytochrome c, while reducing the levels of Mcl-1 and survivin in osteosarcoma cells. Furthermore, TF3 significantly reduced the average tumor volume in the xenograft model. Overall, this study suggests that TF3 induces apoptosis in osteosarcoma cells, primarily by regulating the caspase pathway.

## Introduction

Osteosarcoma is a solid tumor that primarily grows in bone tissue and originates from primitive mesenchymal cells 1. Clinically, osteosarcoma is classified as high-grade central, high-grade surface, low-grade central, periosteal, parosteal, or secondary tumor 2. Osteosarcoma has traditionally been treated with chemotherapy and surgery 3. Despite aggressive interventions, the overall prognosis remains poor, with a 5-year survival rate of < 20% in patients with metastasis 4, 5.

Inducing apoptosis in neoplastic cells has been explored as a potential therapeutic strategy for managing osteosarcoma 6-11. For example, previous studies have shown that the curcumin analogs GO-Y030 and HO-3867 effectively trigger both extrinsic and intrinsic apoptotic pathways in human osteosarcoma cells 9, 11. *In vitro* evidence suggests that GO-Y078 reduces proliferation and induces apoptosis in osteosarcoma cells through the activation of JNK and p38 signaling pathways 10. Additionally, deoxyshikonin has been shown to induce apoptosis in osteosarcoma cells by regulating the p38 pathway 12. Therefore, understanding the molecular mechanisms of apoptosis is crucial for developing targeted therapies for osteosarcoma.

Theaflavin-3,3′-digallate (TF3), a member of the flavin family, is involved in various proliferation pathways. TF3 reduced erastin-induced ferroptosis in the chondrocytes of patients with osteoarthritis 13. TF3 also inhibited the proliferation of ovarian cancer stem cells by suppressing the Wnt/β-Catenin pathway 14. Few studies have analyzed the effects of TF3 on the growth of osteosarcoma cells. Given that deoxyshikonin arrests the cell cycle to inhibit the growth of osteosarcoma cells 12, TF3 can be expected to induce apoptosis in these cells, warranting further investigation. The present study investigated the effects of TF3 on the growth and apoptosis of osteosarcoma cells. The study also explored key apoptotic pathways activated by TF3.

## Materials and Methods

### Materials

Eagle's Minimum Essential Medium, Dulbecco's Modified Eagle Medium, and fetal bovine serum were obtained from Gibco Life Technologies. Antibodies against cleaved caspase-3, cleaved caspase-9, phosphorylated p53, phosphorylated H2Ax, Bax, Bak1, cytochrome c, Mcl-1, and survivin were acquired from Cell Signaling Technology. A Human Apoptosis Array Kit and a fluorescein isothiocyanate (FITC) Annexin V Apoptosis Detection Kit I were purchased from R&D Systems and BD Biosciences, respectively. Other reagents such as JC-1 and N-acetyl-L-cysteine (NAC) were sourced from Sigma-Aldrich. TF3 (purity, ≥95%; high-performance liquid chromatography grade) was obtained from ChemFaces and dissolved in dimethyl sulfoxide (Sigma-Aldrich).

### Cell culture and TF3 treatment

The osteosarcoma cell lines used in this study were 143B (derived from a 13-year-old girl) and U2OS (derived from a 15-year-old girl). These cells were obtained from the Food Industry Research and Development Institute (Hsinchu, Taiwan). The 143B cells were cultured in Eagle's Minimum Essential Medium, and the U2OS cells were cultured in Dulbecco's Modified Eagle Medium 15. Both media were supplemented with 10% fetal bovine serum, 1% penicillin/streptomycin, and 5 mL of glutamine. The cells were maintained at 37 °C in a humidified incubator containing 5% CO_2_. After incubation, the cells were treated with various concentrations of TF3 (50, 75, and 100 μM).

### MTT assay

A 3-(4,5-dimethylthiazol-2-yl)-2,5-diphenyltetrazolium bromide (MTT) assay was performed to assess the effect of TF3 on the viability of 143B and U2OS cells 16. The cells were seeded into 24-well plates (cell density: 4 × 10^4^/well) and incubated for approximately 16 h before TF3 treatment at 37 °C for 24 or 48 h. After treatment, the MTT assay was performed as described in other studies 15, 16. NAC and caspase inhibitor carbobenzoxy-valyl-alanyl-aspartyl-[O-methyl]-fluoromethylketone (Z-VAD-FMK) were used in specific MTT assays.

### Flow cytometry and annexin V-FITC staining

For further experiments, 143B and U2OS cells were cultured in 6-cm plates (cell density: 2 × 10⁵/well) and treated with TF3 (concentrations: 0, 50, 75, and 100 μM) for 24 or 48 h. Subsequently, the cells, including floating nonviable cells, were harvested through trypsinization and stained with propidium iodide. After staining, the cells were transferred to Eppendorf tubes (cell density: 2 × 10⁵/tube) to analyze the cell cycle and measure reactive oxygen species (ROS) by using a dihydroethidium assay kit and a flow cytometer. To investigate the apoptotic effects of TF3, annexin V-FITC staining was performed following the relevant kit manufacturer's protocol. The results of annexin V-FITC staining and propidium iodide staining were used to differentiate between apoptosis and necrosis 17.

### Measurement of mitochondrial membrane potential

To assess the stability of osteosarcoma cells, the mitochondrial membrane potential (MMP) of TF3-treated 143B and U2OS cells was measured. JC-1, a stain that detects MMP imbalance, was used for this analysis 18.

### Western blotting

The apoptotic pathways activated by TF3 in osteosarcoma cells were identified through Western blotting. For this, 143B and U2OS cells were seeded in 6-cm plates (cell density: 2 × 10^5^/well) and incubated for 16 h. The cells were then treated with TF3 for 24 h. Total cell lysates were prepared. Western blotting was performed using primary antibodies against cleaved caspase-3, cleaved caspase-9, phosphorylated p53, phosphorylated H2Ax, Bax, Bak1, cytochrome c, Mcl-1, and survivin. The blots were subsequently incubated with horseradish peroxidase-conjugated goat antirabbit or antimouse immunoglobulin G for 1 h. The intensity of each protein band was quantified using densitometry 19.

### *In vivo* experiments

To investigate the effect of TF3 on the growth of osteosarcoma cells *in vivo*, male nude mice (BALB/c AnN.Cg-Foxnnu/Crl Narl; age: 4 to 5 weeks) were obtained from BioLASCO (Taipei City, Taiwan). The mice were maintained under a regular 12-h light/dark cycle and provided with a standard rodent diet (Laboratory Rodent Diet 5001; LabDiet, St. Louis, MO, USA). A xenograft mouse model was established by injecting the mice (into the proximal tibia) with 143B osteosarcoma cells (density: 5 × 10^5^ to 1 × 10^6^ cells in 70 μL of phosphate-buffered saline) in 30 μL of Matrigel. Then, the mice were fed TF3 at experimental concentrations (10 and 20 mg/kg) thrice a week. Body weight and tumor size (measured using a luciferase bioluminescence kit and a vernier caliper) were recorded every 3 days over the 32-day treatment period. Tumor size was calculated as follows: 0.5 × long diameter × short diameter × short diameter. After euthanasia, tumor tissues were harvested for volume analysis and immunohistochemical staining (to detect Ki-67 expression under a light microscope; ×1000). All animal protocols and experimental procedures were approved by the Institutional Animal Care and Use Committee of Chung Shan Medical University, Taichung, Taiwan (Approval No. 2623).

### Statistical analysis

The independent *t* test was used for comparisons between two groups. One-way analysis of variance with post hoc Tukey's test was used for comparisons involving > 2 groups. Statistical significance was set at *P* < 0.05.

## Results

### Viability of osteosarcoma cells after TF3 treatment

**Figure 1:**
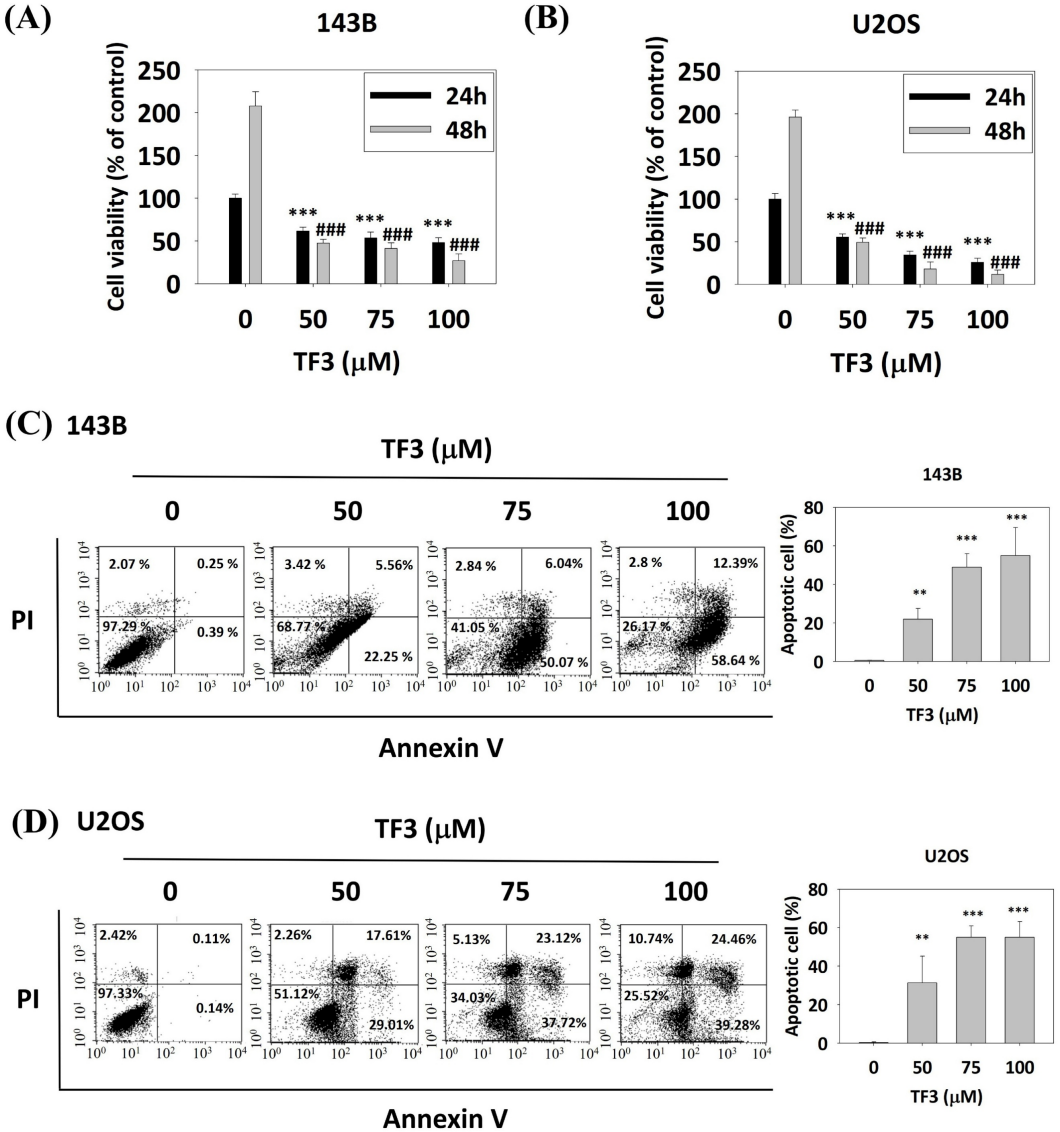
** The cell viability of osteosarcoma cells after TF3 treatment with different analysis.** (A) The cell viability of 143B osteosarcoma cells after TF3 treatment according to MTT assay. (B) The cell viability of U2OS osteosarcoma cells after TF3 treatment according to MTT assay. (C) The apoptotoic effect of 143B osteosarcoma cells after TF3 treatment according to PI/AnnexinV flow cytometry. (D) The apoptotoic effect of U2OS osteosarcoma cells after TF3 treatment according to PI/AnnexinV flow cytometry. **P < 0.01 as compared with DMSO treatment (the 0 μM). ***P < 0.001 as compared with DMSO treatment (the 0 μM). ^###^*P* < 0.001 as compared with DMSO treatment (the 0 μM).

Treatment with 50, 75, and 100 μM TF3 significantly reduced the viability of 143B (Figure 1A) and U2OS (Figure 1B) cells compared with that of control cells (all *P* < 0.05). Flow cytometry after annexin V-FITC staining indicated higher levels of apoptosis in both 143B (Figure 1C) and U2OS (Figure 1D) cells treated with 50, 75 or 100 μM TF3 than in control cells (all *P* < 0.05).

### MMP in osteosarcoma cells

**Figure 2:**
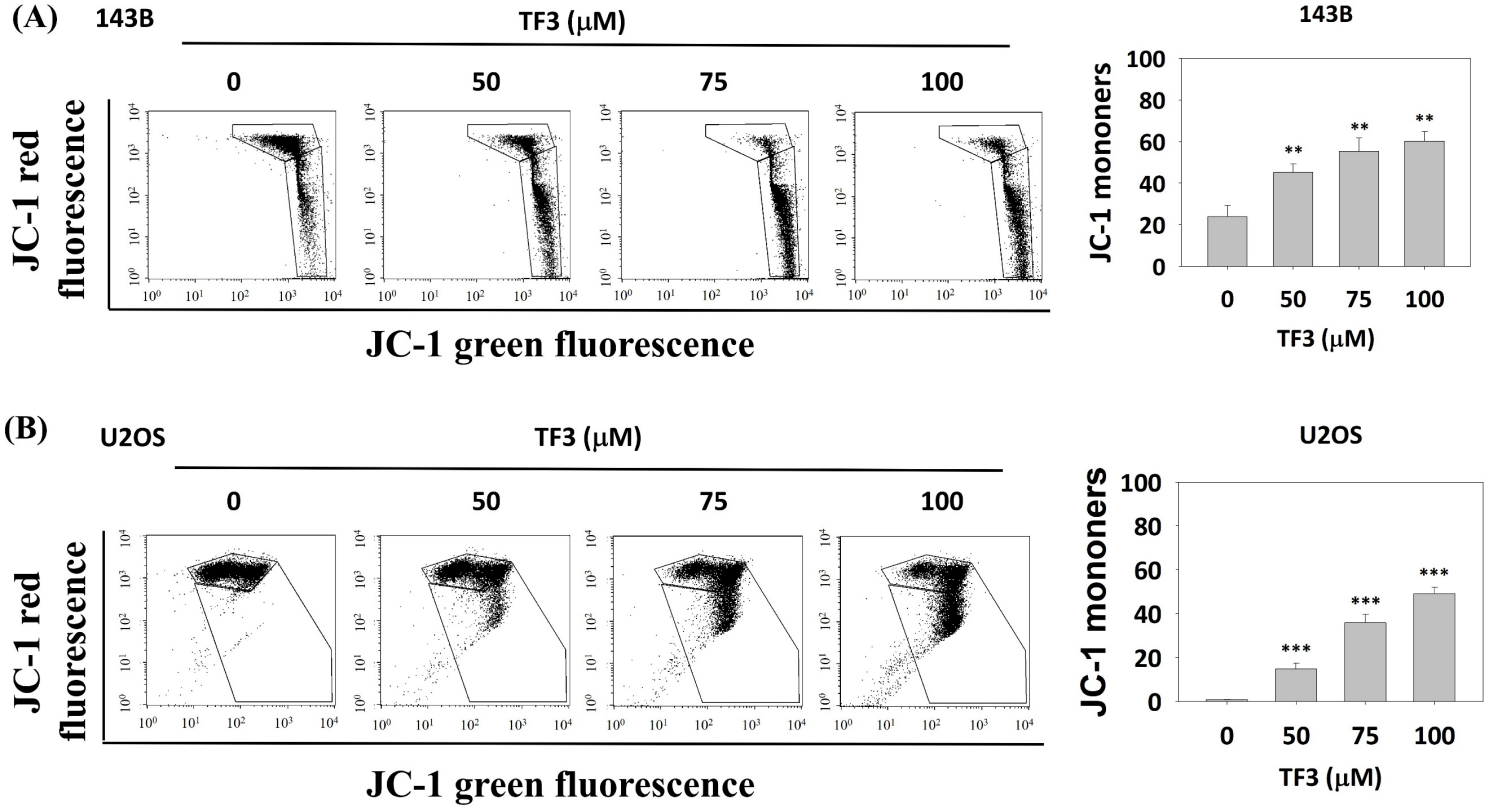
** The mitochondria membrane potential changes of osteosarcoma cells after TF3 treatment.** (A) The mitochondria membrane potential change of 143B osteosarcoma cells after TF3 treatment according to JC-1 green fluorescence stain. (B) The mitochondria membrane potential change of U2OS osteosarcoma cells after TF3 treatment according to JC-1 green fluorescence stain. **P < 0.01 as compared with DMSO treatment (the 0 μM). ***P < 0.001 as compared with DMSO treatment (the 0 μM).

Figure 2 presents the results of JC-1 staining in osteosarcoma cells treated with 50, 75 and 100 μM of TF3. The results revealed higher rates of MMP imbalance in 143B (Figure 2A) and U2OS (Figure 2B) cells treated with 50, 75, or 100 μM TF3 than in control cells (all *P* < 0.05).

### ROS in TF3-treated osteosarcoma cells

**Figure 3:**
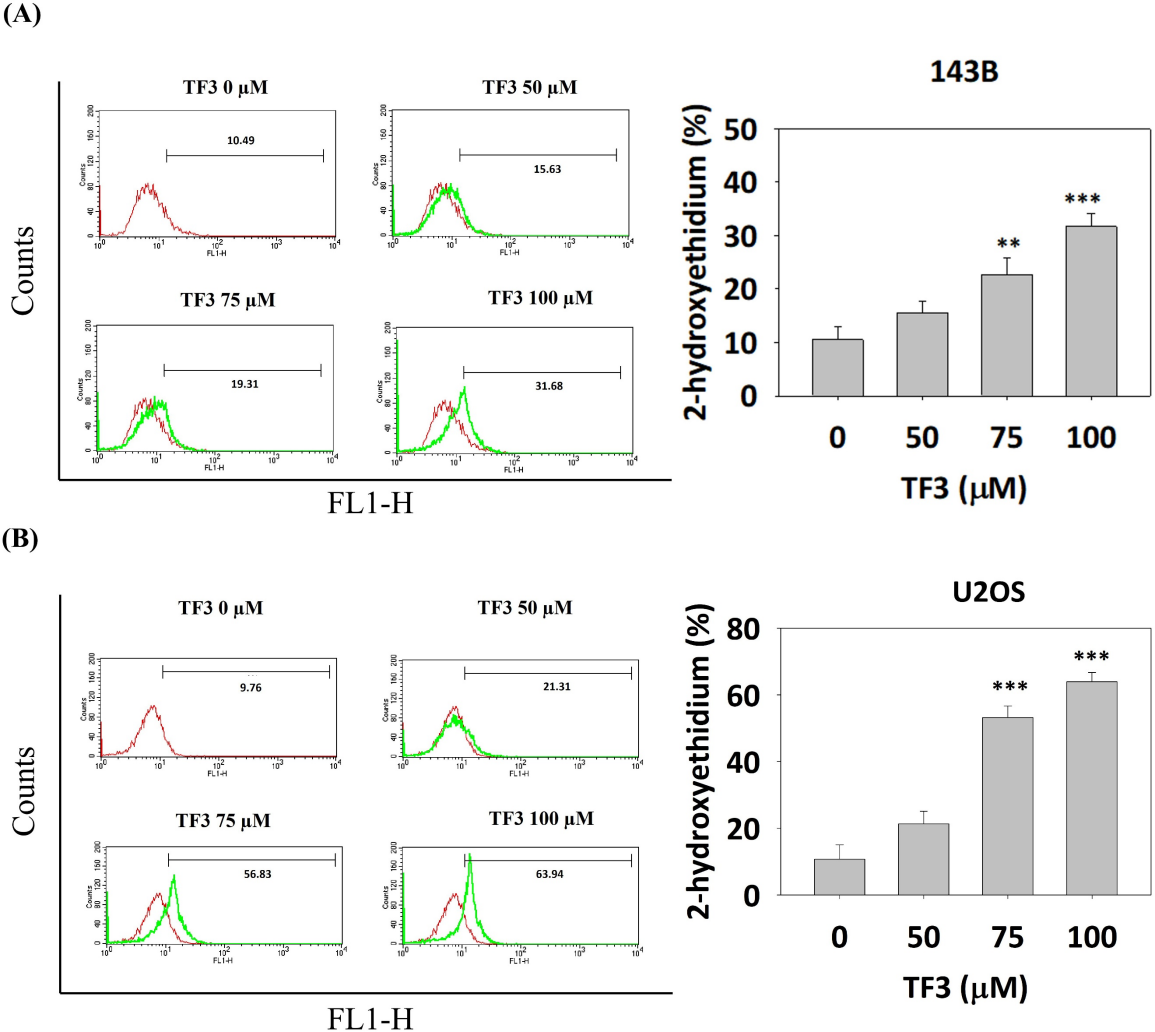
** The level of reactive oxygen species in osteosarcoma cells after TF3 treatment.** (A) The reactive oxygen species amount of 143B osteosarcoma cells after TF3 treatment according to DHE assay. (B) The reactive oxygen species amount of U2OS osteosarcoma cells after TF3 treatment according to DHE assay. **P < 0.01 as compared with DMSO treatment (the 0 μM). ***P < 0.001 as compared with DMSO treatment (the 0 μM).

**Figure 4:**
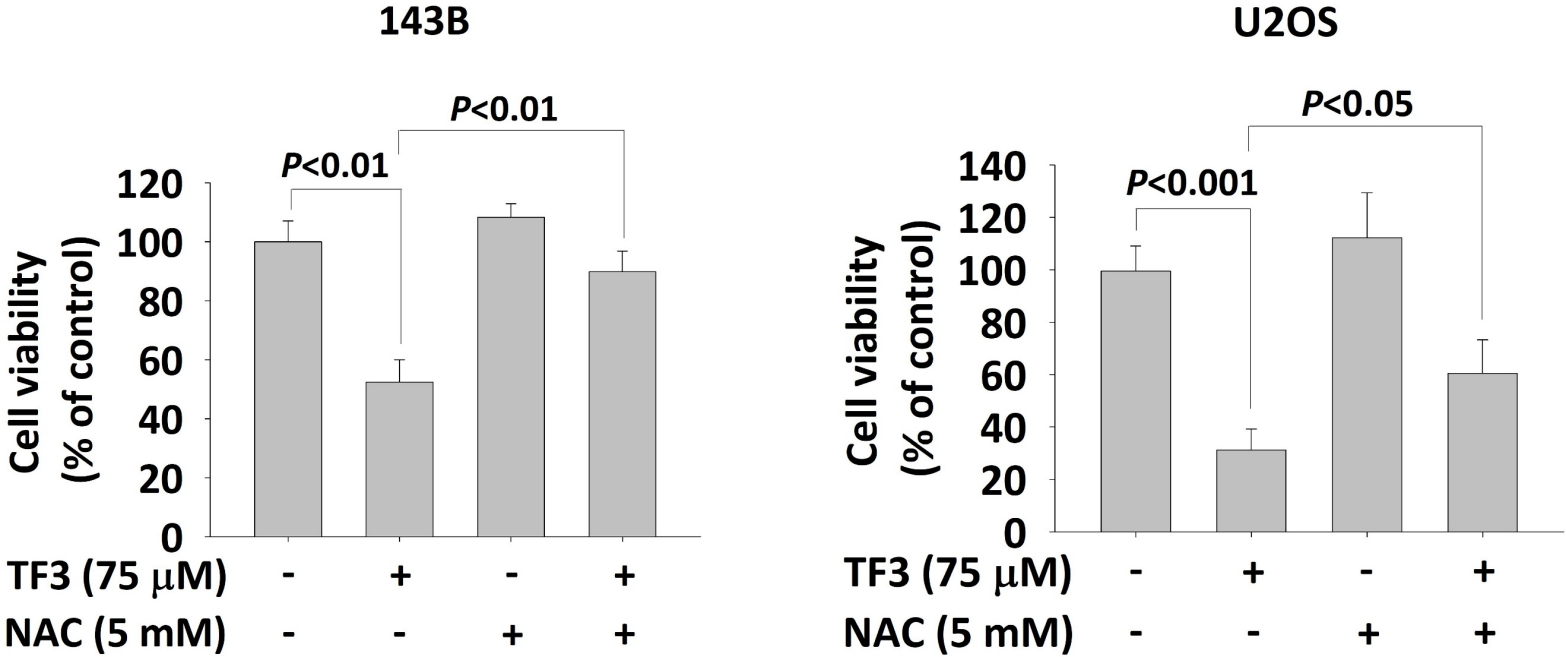
** The cell viability with N-acetyl-L-cysteine treatment of osteosarcoma cells after TF3 treatment.** (A) The cell viability with or without NAC treatment of 143B osteosarcoma cells after TF3 treatment according to MTT assay. (B) The cell viability with or without NAC treatment of U2OS osteosarcoma cells after TF3 treatment according to MTT assay.

ROS distributions, analyzed using a dihydroethidium assay, indicated significantly higher levels of 2-hydroxyethidium in 143B (Figure 3A) and U2OS (Figure 3B) cells treated with 75 or 100 μM TF3 than in control cells (both *P* < 0.05). The MTT assay confirmed lower viability in TF3-treated 143B and U2OS cells than in control cells (both *P* < 0.05). Moreover, treatment with NAC, an antioxidant, significantly reversed cell viability after TF3 treatment (both *P* < 0.05; Figure 4).

### Apoptotic pathway in TF3-treated osteosarcoma cells

**Figure 5:**
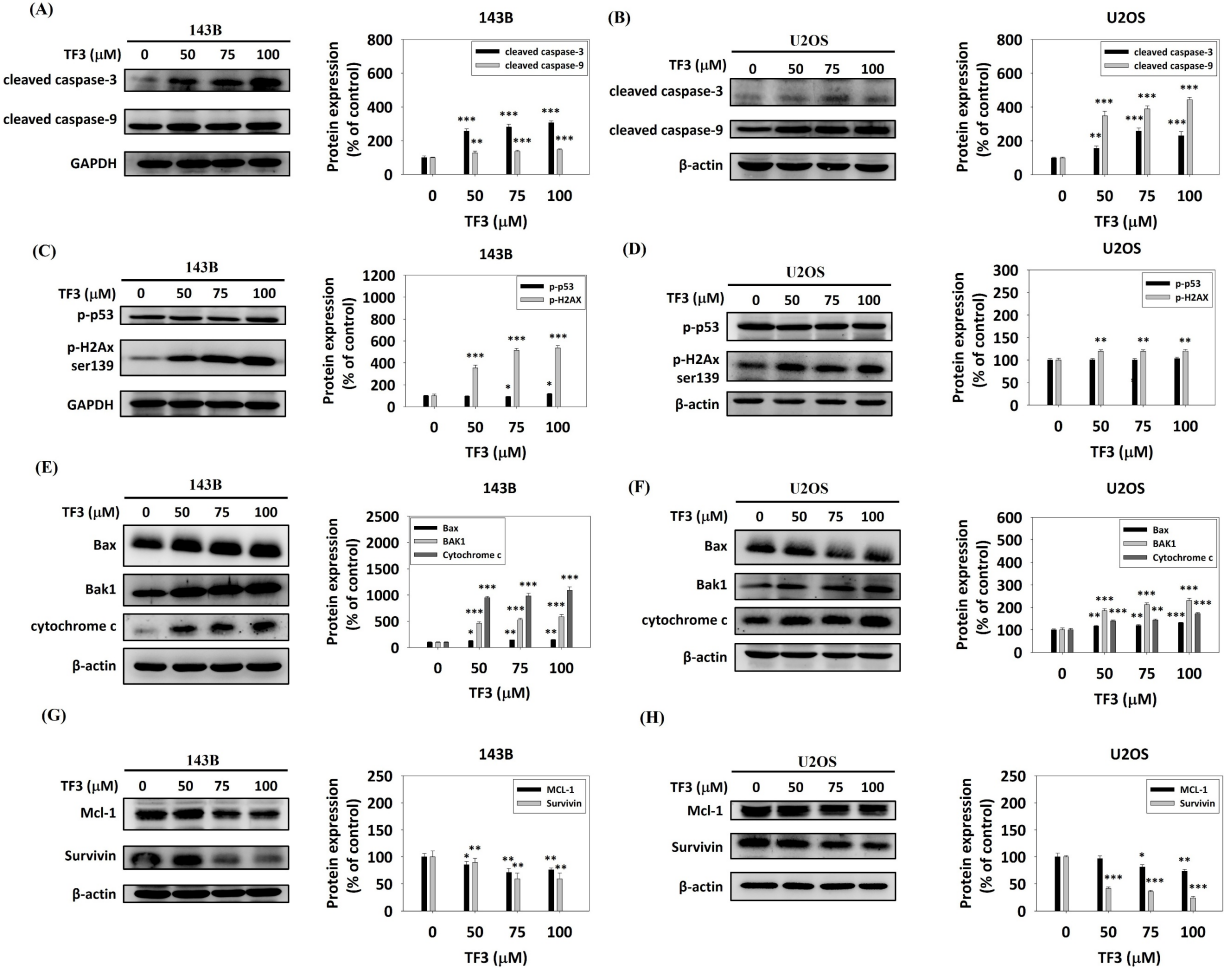
** The apoptotic pathway of osteosarcoma cells after TF3 treatment according to Western blotting analysis.** (A) The expression of cleaved-caspase-3 and -9 of 143B osteosarcoma cells after TF3 treatment. (B) The expression of cleaved-caspase-3 and 9 of U2OS osteosarcoma cells after TF3 treatment. (C) The expression of phosphorylated p53 and phosphorylated H2Ax of 143B osteosarcoma cells after TF3 treatment. (D) The expression of phosphorylated p53 and phosphorylated H2Ax of U2OS osteosarcoma cells after TF3 treatment. (E) The expression of Bax, Bak1 and cytochrome C of 143B osteosarcoma cells after TF3 treatment. (F) The expression of Bax, Bak1 and cytochrome C of U2OS osteosarcoma cells after TF3 treatment. (G) The expression of Mcl-1 and survinin of 143B osteosarcoma cells after TF3 treatment. (H) The expression of Mcl-1 and survinin of U2OS osteosarcoma cells after TF3 treatment. *P < 0.05 as compared with DMSO treatment (the 0 μM). **P < 0.01 as compared with DMSO treatment (the 0 μM). ***P < 0.001 as compared with DMSO treatment (the 0 μM).

Western blotting revealed significant upregulation of cleaved caspase-3 and cleaved caspase-9 in 143B (Figure 5A) and U2OS (Figure 5B) cells treated with 50, 75, or 100 μM TF3 compared with the levels in control cells (all *P* < 0.05). The levels of phosphorylated p53 and phosphorylated H2Ax were significantly higher in 143B cells (Figure 5C). Additionally, treatment with 50, 75, or 100 μM TF3 led to a significant upregulation of phosphorylated H2Ax in U2OS cells compared to control cells (all *P* < 0.05) (Figure 5D). The levels of Bax, Bak1, and cytochrome c were significantly higher in 143B (Figure 5E) and U2OS (Figure 5F) cells treated with 50, 75 or 100 μM TF3 than in control cells (all *P* < 0.05). The level of Mcl-1 and survivin significantly decreased in 143B (Figure 5G) and U2OS (Figure 5H) cells treated with 50, 75, or 100 μM TF3 compared with the levels in control cells (all *P* < 0.05).

### Interaction between Z-VAD-FMK and TF3 in osteosarcoma cells

**Figure 6:**
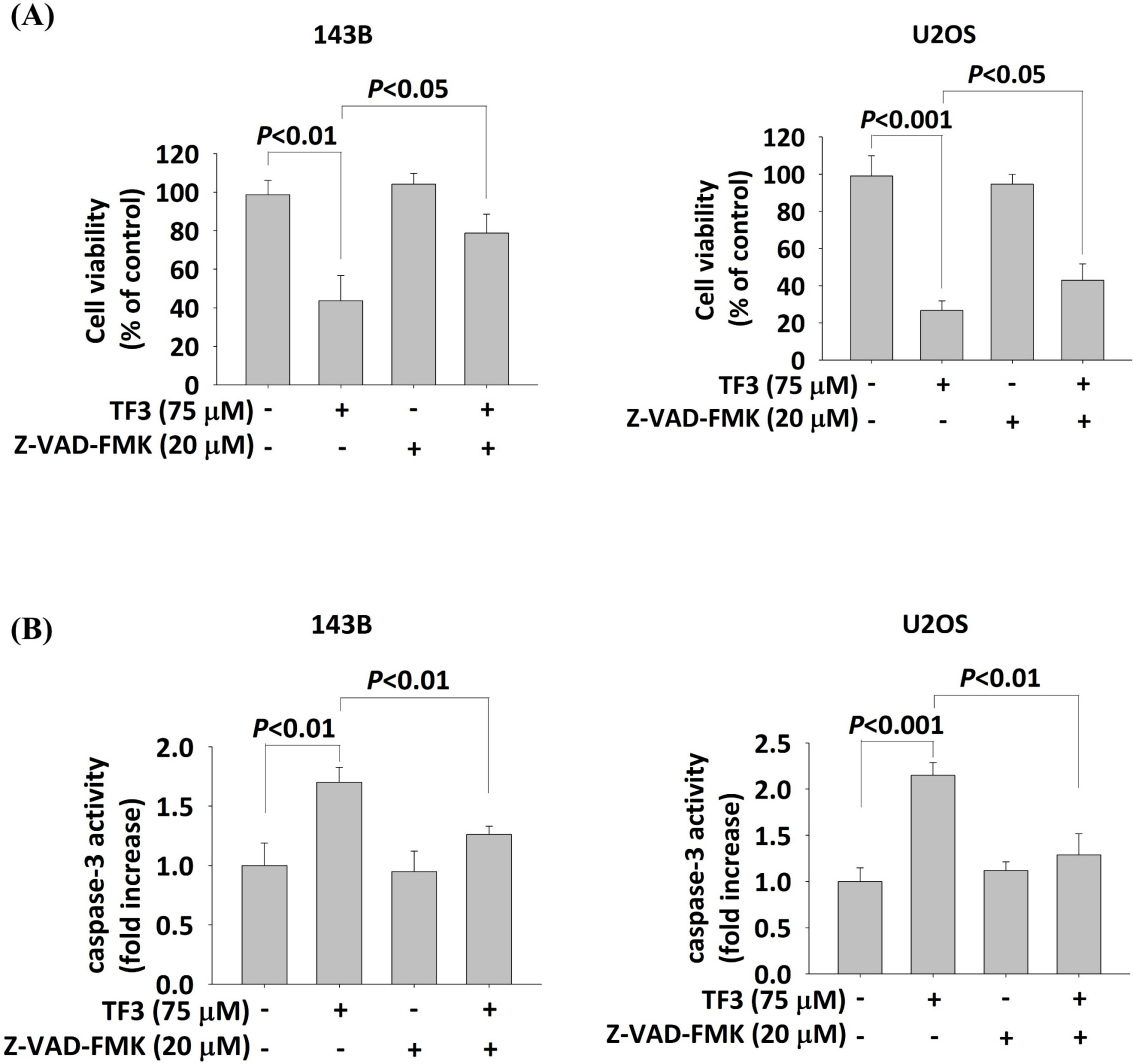
** The cell viability and caspase-3 activity with caspase inhibitor treatment of osteosarcoma cells after TF3 treatment.** (A) The cell viability with or without caspase inhibitor Z-VAD-FMK treatment of 143B and U2OS osteosarcoma cells after TF3 treatment according to MTT assay. (B) The caspase-3 activity with or without caspase inhibitor Z-VAD-FMK treatment of 143B and U2OS osteosarcoma cells after TF3 treatment according to MTT assay.

Treatment with 75 μM TF3 significantly reduced the viability of both 143B and U2OS cells (both *P* < 0.05). However, the treatment with Z-VAD-FMK (20 μM) significantly increased the viability of TF3-treated 143B and U2OS cells (both *P* < 0.05; Figure 6A). Caspase-3 activity was elevated in 143B and U2OS cells treated with 75 μM TF3 (both *P* < 0.05). The addition of Z-VAD-FMK significantly mitigated this activity (both *P* < 0.05; Figure 6B).

### Effect of TF3 treatment on the xenograft model

**Figure 7:**
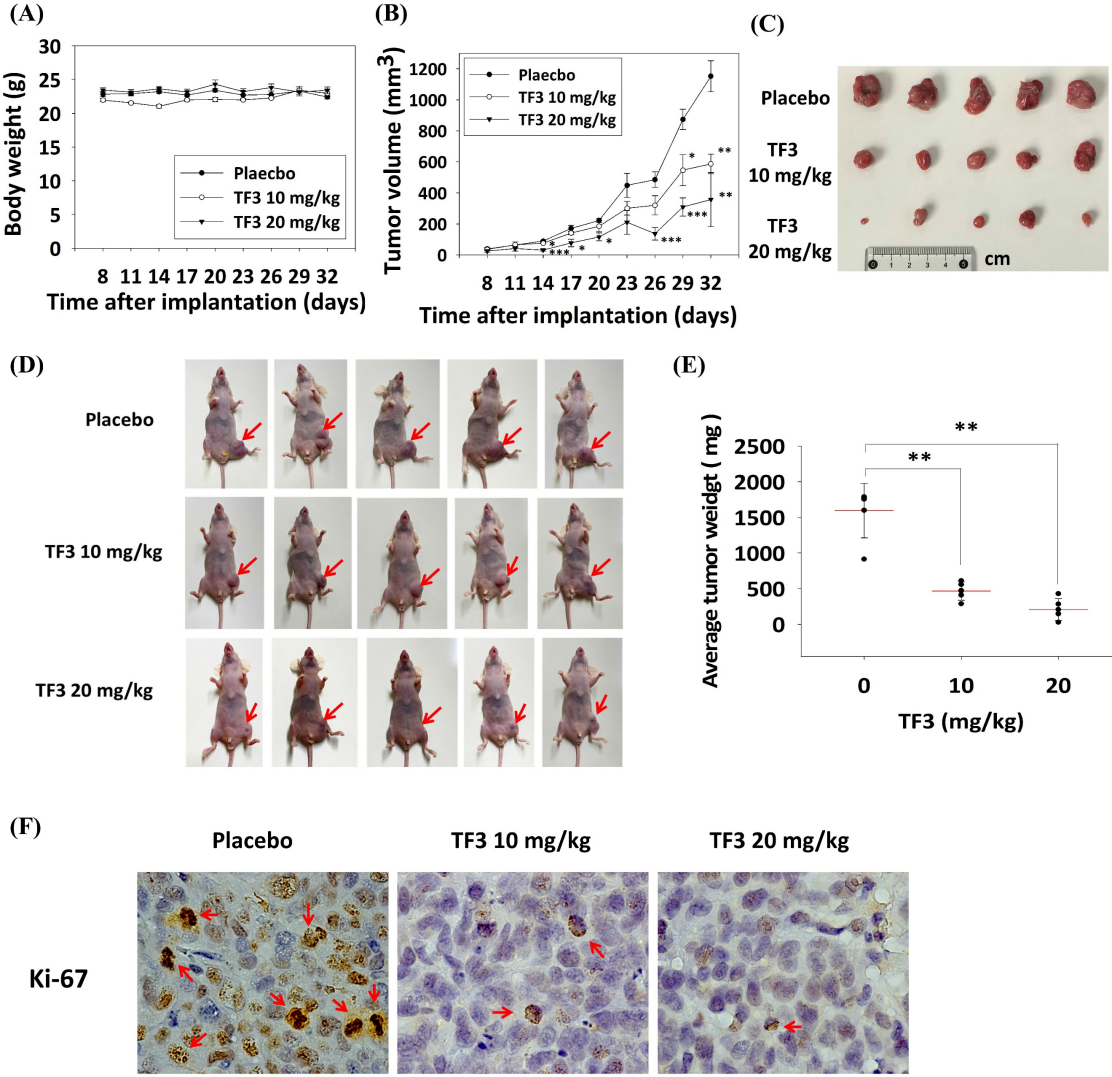
** The xenograft model for the osteosarcoma proliferation in mice after TF3 treatment.** (A) The average body weight change of mice after the xenograft of 143B osteosarcoma cells and TF3 treatment. (B) The average tumor volume change of mice after the xenograft of 143B osteosarcoma cells and TF3 treatment. (C) The gross appearance of 143B osteosarcoma tumor after TF3 treatment. (D) The appearance of 143B osteosarcoma tumor in mice after TF3 treatment. (E) The average tumor weight of mice after the xenograft of 143B osteosarcoma cells and TF3 treatment. (F) The Ki-67 expression of mice after the xenograft of 143B osteosarcoma cells and TF3 treatment. **P < 0.01 as compared with placebo group. ***P < 0.001 as compared with placebo group (the 0 μM).

*In vivo* experiments indicated no significant difference in body weight among mice treated with 10 mg/kg TF3, mice treated with 20 mg/kg TF3, and control mice (*P* > 0.05; Figure 7A). However, the average tumor volume was significantly lower in mice treated with 10 or 20 mg/kg TF3 than in control mice (both *P* < 0.05; Figure 7B). Images of osteosarcomas are presented in Figure 7C, and their appearance in mice is depicted in Figure 7D. Tumor weight was significantly lower in mice treated with 10 or 20 mg/kg TF3 than in control mice (both *P* < 0.05; Figure 7E). Furthermore, the level of Ki-67 expression, indicative of tumor proliferation, was lower in mice treated with 10 or 20 mg/kg TF3 than in control mice (Figure 7F).

## Discussion

This study demonstrated that TF3 treatment significantly reduced cell viability, increased MMP imbalance, and elevated ROS levels in osteosarcoma cells. The treatment upregulated cleaved caspase-3, cleaved caspase-9, phosphorylated H2Ax, Bax, Bak1, and cytochrome c, while downregulating survivin and Mcl-1 in osteosarcoma cells. Notably, the addition of Z-VAD-FMK, a caspase inhibitor, increased cell viability after TF3 treatment. In the xenograft mouse model, TF3 treatment reduced tumor volume, tumor weight, and Ki-67 expression.

Patients with osteosarcoma typically receive a guarded prognosis. Osteosarcoma is influenced by several pathways and factors 5, 11, 20. The protein kinase B and extracellular signal-regulated kinase pathways promote the growth of osteosarcoma cells 3. The receptor activator of nuclear factor-κB pathway facilitates bone resorption and osteosarcoma formation 21. Doxorubicin, cisplatin, and methotrexate have been used to treat osteosarcoma 1. Tumor lysate-pulsed dendritic cells were used to reduce the proliferation of osteosarcoma cells in mice 20. Inhibiting N4-acetylcytidine acetyltransferase 10 can inhibit the progression of osteosarcoma 22. Apoptosis is a crucial pathway for inhibiting the formation of osteosarcoma 11, 12. HO-3867 increased the rate of apoptosis in an experimental model of osteosarcoma 11. Deoxyshikonin reduced the viability of osteosarcoma cells by altering the p38, c-Jun N-terminal kinase, and extracellular signal-regulated kinase pathways 12. TF3, a substance with anti-inflammatory and antioxidant properties 23, 24, induced apoptosis and autophagy in several disease models 23. TF3 promotes apoptosis in prostate cancer cell via the protein kinase Cδ/acid sphingomyelinase pathway 25. Given that osteosarcoma can be inhibited by inducing apoptosis and that TF3 induces apoptosis in other neoplasms 12, 14, we hypothesized that TF3 treatment would induce apoptosis in experimental models of osteosarcoma. This hypothesis is supported by our findings.

TF3 treatment reduced viability in two osteosarcoma cell lines. Chalcones induce apoptosis in osteosarcoma cells 26. Oridonin promotes apoptosis in 143B and U2OS cells 27. Few studies have investigated the effect of TF3 on the apoptosis of osteosarcoma cells. Our study provides preliminary evidence of TF3's effect on the apoptosis of these cells. We used MTT assay and flow cytometry methods to analyze the effect of TF3 on apoptosis in osteosarcoma cells. Both methods are reliable for confirming cell viability. We demonstrated that TF3 treatment induces apoptosis in osteosarcoma cells. The MTT assay revealed that viability was the lowest in 143B and U2OS cells treated with 100 μM TF3 and highest in control cells. Flow cytometry indicated that the level of apoptosis was highest in 143B and U2OS cells treated with 100 μM TF3 and lowest in the control cells. These findings indicate that the rate of apoptosis in osteosarcoma cells is positively correlated with the concentration of TF3. Furthermore, MMP imbalance, an indicator of apoptosis 28, was prevalent in TF3-treated 143B and U2OS cells, corroborating the results of the MTT assay and flow cytometry.

TF3 treatment significantly elevated ROS levels in 143B and U2OS cells. Specifically, ROS levels were significantly higher in cells treated with 75 or 100 μM TF3 than in control cells. This finding indicates that the effect of TF3 on ROS levels in osteosarcoma cells is positively correlated with the concentration of TF3. ROS is an indicator of apoptosis 29. Elevated ROS levels induce apoptosis in renal cancer cells 29. Evidence suggests that tripartite motif-containing protein 22 induces apoptosis in osteosarcoma cells by increasing ROS levels 30. Deoxyelephantopin increases ROS levels and induces apoptosis in osteosarcoma cells 31. ROS inducers inhibit proliferation and promote apoptosis in osteosarcoma cells 32. Curcumin induces apoptosis in osteosarcoma cells by elevating ROS levels 33. Therefore, the increased ROS levels in TF3-treated osteosarcoma cells may indicate the initiation of apoptosis. In the present study, NAC (an ROS suppressor and antioxidant) increased the viability of osteosarcoma cells even after TF3 treatment. This finding suggests a correlation between ROS levels and TF3-induced apoptosis in osteosarcoma cells.

Western blot analysis revealed an upregulation of cleaved caspase-3 and cleaved caspase-9 in 143B and U2OS cells. The caspase family is involved in apoptosis 34, 35. Procaspase-3 and procaspase-9 are cleaved into their active forms, cleaved caspase-3 and cleaved caspase-9, respectively, leading to apoptosis in several human cancers, including head and neck cancer 36. Increased levels of cleaved caspase-3 and cleaved caspase-9 in TF3-treated osteosarcoma cells further suggest that TF3 induces apoptosis in these cells. In addition to the caspase family members, proteins such as phosphorylated p53, phosphorylated H2Ax, Bax, Bak1, cytochrome c, Mcl-1, and survivin mediate apoptosis in human cells 37-40. Elevated levels of these proteins, except for survivin, in TF3-treated osteosarcoma cells supports the role of TF3 in inducing apoptosis. Notably, treatment with 50 μM TF3 significantly altered the levels of the aforementioned proteins in osteosarcoma cells; this finding suggests that low concentrations of TF3 can induce apoptosis, but high concentrations of TF3 (>75 μM) are required to cause cell death. Treatment with Z-VAD-FMK increased viability and reduced caspase-3 activity in TF3-treated cells; this finding suggests that TF3 induces apoptosis in osteosarcoma cells via the caspase-3 pathway.

In the xenograft model, TF3 treatment significantly reduced both tumor volume and weight. Few studies have reported such findings. One study demonstrated that trabectedin reduced tumor volume in a xenograft model of osteosarcoma 41. Given that trabectedin induces apoptosis 42, TF3 can be expected to reduce tumor parameters in xenograft models. Notably, TF3 exerted no significant effect on the average body weight of mice; this finding highlights the safety of TF3 treatment in mice with osteosarcoma. Furthermore, TF3 downregulated the expression of Ki-67, an indicator of cell proliferation. A high level of Ki-67 expression is correlated with the progression of colorectal adenocarcinoma 43. Thus, the downregulated Ki-67 expression in TF3-treated mice further supports the anticancer effects of TF3. Therefore, TF3 holds promise for reducing tumor size in animal models of osteosarcoma.

**Figure 8:**
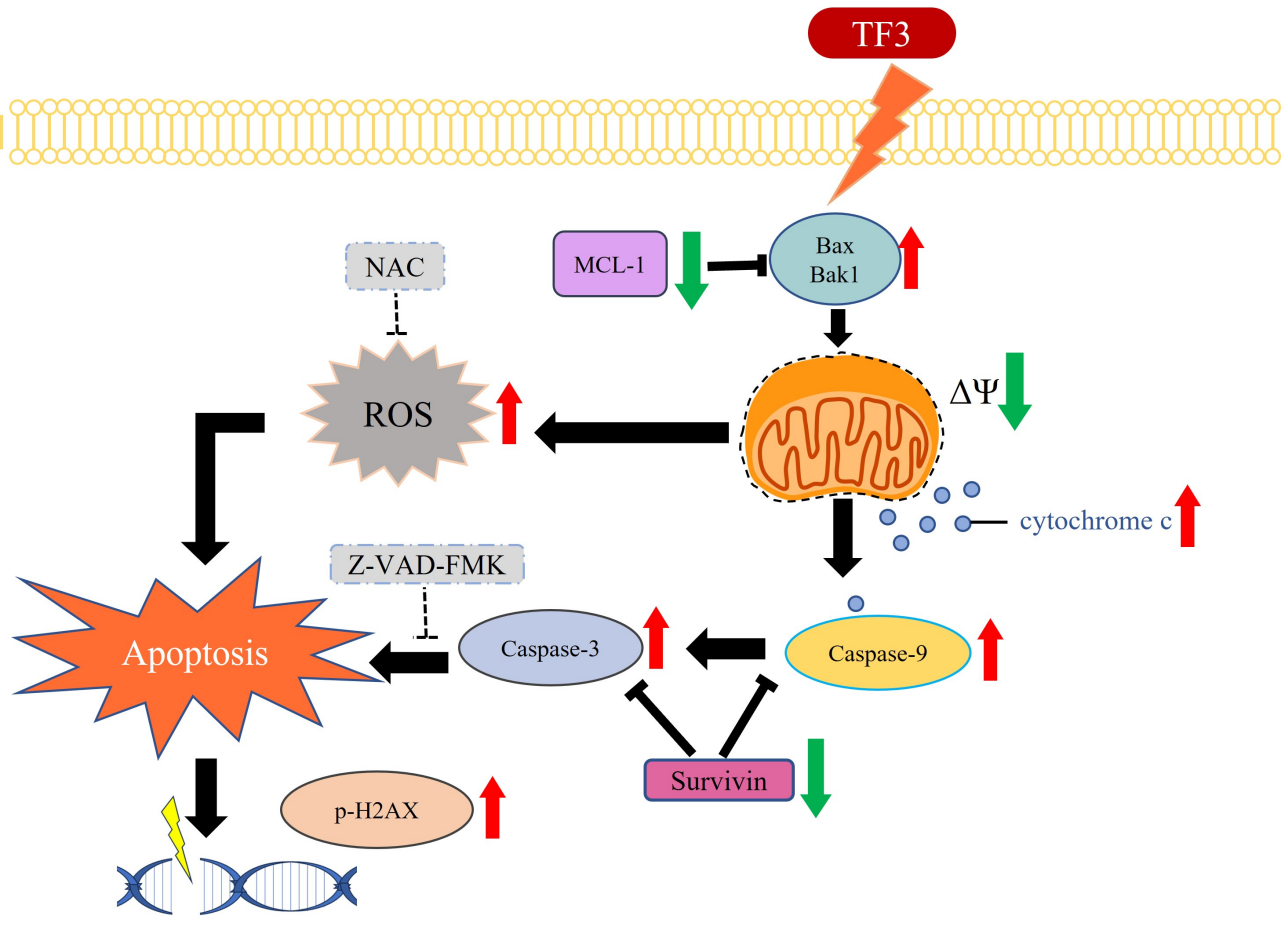
The schematic graph of TF3-related pathway for apoptosis in osteosarcoma.

In conclusion, TF3 treatment significantly reduces the viability of osteosarcoma cells. Furthermore, TF3-induced apoptosis in osteosarcoma cells is associated with the caspase-3 pathway (Figure 8). TF3 treatment also reduced tumor size in mice with osteosarcoma. Further large-scale studies are required to confirm the efficacy of TF3 in inhibiting the growth osteosarcoma cells in human patients.
